# Causes of death among women of reproductive age during the war in Tigray, Ethiopia

**DOI:** 10.1371/journal.pone.0299650

**Published:** 2024-03-13

**Authors:** Hiluf Ebuy Abraha, Hale Teka, Awol Yemane Legesse, Mohamedawel Mohamedniguss Ebrahim, Mache Tsadik, Girmatsion Fisseha, Bereket Berhe, Brhane Ayele, Gebrehaweria Gebrekurstos, Tesfit Gebremeskel, Tsega Gebremariam, Martha Yemane Hadush, Tigist Hagos, Abraha Gebreegziabher, Kibrom Muez, Haile Tesfay, Hagos Godefay, Afework Mulugeta

**Affiliations:** 1 College of Health Science, Mekelle University, Mekelle, Tigray, Ethiopia; 2 Arnold School of Public Health, University of South Carolina, Columbia, South Carolina, United States of America; 3 Tigray Health Research Institute, Mekelle, Tigray, Ethiopia; 4 Tigray Regional Health Buruea, Mekelle, Tigray, Ethiopia; Haramaya University Faculty of Health Sciences: Haramaya University College of Health and Medical Sciences, ETHIOPIA

## Abstract

**Background:**

In resource-limited countries with weak healthcare systems, women of reproductive age are particularly vulnerable during times of conflict. In Tigray, Ethiopia, where a war broke out on 04 November 2020, there is a lack of information on causes of death (CoD) among women of reproductive age. This study aims to determine the underlying CoD among women of reproductive age during the armed conflict in Tigray.

**Methods:**

This community-based survey was carried out in six Tigray zones, excluding the western zone for security reasons. We used a multistage stratified cluster sampling method to select the smallest administrative unit known as Tabiya. Data were collected using a standardized 2022 WHO Verbal Autopsy (VA) tool. The collected data were analyzed using the InterVA model using R analytic software. The study reported both group-based and cause-specific mortality fractions.

**Results:**

A total of 189,087 households were screened and 832 deaths were identified among women of reproductive age. The Global Burden of Disease classification showed that infectious and maternal disorders were the leading CoD, accounting for 42.9% of all deaths. External causes contributed to 26.4% of fatalities, where assault accounted for 13.2% of the deaths. Maternal deaths made up 30.0% of the overall mortality rate. HIV/AIDS was the primary CoD, responsible for 13.2% of all deaths and 54.0% of infectious causes. Other significant causes included obstetric hemorrhage (11.7%) and other and unspecified cardiac disease (6.6%).

**Conclusions:**

The high proportion of infectious diseases related CoD, including HIV/AIDS, as well as the occurrence of uncommon external CoD among women, such as assault, and a high proportion of maternal deaths are likely the result of the impact of war in the region. This highlights the urgent need for targeted interventions to address these issues and prioritize sexual and reproductive health as well as maternal health in Tigray.

## Background

Women in the reproductive age group are considered a vulnerable population, particularly in resource-limited countries with inadequate healthcare systems. Globally, pregnancy related mortality and HIV/AIDS stand as primary factors contributing to the mortality of women in their reproductive years, with HIV consistently holding the top position for decades [1–3]. Women of reproductive age face additional risks on top of the physiological changes associated with childbirth [4]. Traditional health threats are more prevalent among them, especially during times of war when a country’s healthcare system is severely disrupted. Reproductive age women in low-resource countries are particularly affected by these challenges, as they already have limited access to healthcare, and the conflicts further exacerbate the situation [5–9].

The war erupted in Tigray, Ethiopia on 04 November 2020, leading to a significant humanitarian crisis in the region. Previously recognized for its exemplary healthcare system in the country, Tigray faced a dire situation with only 27.5% of hospitals and 17.5% of health centers operating and providing limited services [10,11]. During the two-year prolonged conflict, women endured numerous unimaginable hardships. Widespread instances of sexual and gender-based violence were observed [12], while the risk factors associated with fistula experienced an alarming rise [13]. Furthermore, institutional delivery rate declined by 40.0% [14] and many obstetric and gynecologic services including gynecological oncology services stopped [15,16].

Despite extensive documentation of the devastating impact of the war in Tigray, previous studies have not explored the underlying causes that contribute to mortality. While it is widely acknowledged that the conflict has claimed many lives [17,18], there is a significant research gap on the specific causes of these deaths, both direct and indirect. This study, aims to determine this gap by systematically examining and assigning the most probable underlying CoD among women of reproductive age, shedding light on the comprehensive impact of the war on mortality in Tigray. Using Verbal Autopsy (VA) based cause of death (CoD) assignment, this research will provide a more comprehensive understanding of the complex consequences of the conflict on reproductive age women in Tigray.

## Methods

### Study area and setting

The study was conducted in Tigray, northern Ethiopia, which has 7 zones with 93 districts. In Tigray region, the average household size is 4.2 people [19], with household size in rural areas higher than urban. For security reasons, the study did not include the western zone and some districts of the northwest and eastern zones. The study period covered November 4, 2020-May 8, 2022, during an active war period in the region.

### Study design and participants

This post hoc analysis utilized data initially gathered to investigate the causes of maternal mortality [20]. Subsequent to the successful completion of the primary study on maternal death, we employed the collected data for a secondary investigation, specifically examining the causes of death among women of reproductive age. A community-based survey was conducted to ascertain the underlying CoD of reproductive age group (12–49 years) women using the standard 2022 WHO VA tool. The study included all deaths of women in the reproductive age group from November 2020 to May 2022 in the Tigray region. Participant recruitment occurred from May 22, 2022, to July 25, 2022. To maintain homogeneity within the study population, internally displaced women were not included in the study. As a result, they were not counted in the household family size, and VA procedures were not conducted for them.

### Sample size and sampling technique

**Fig 1:**
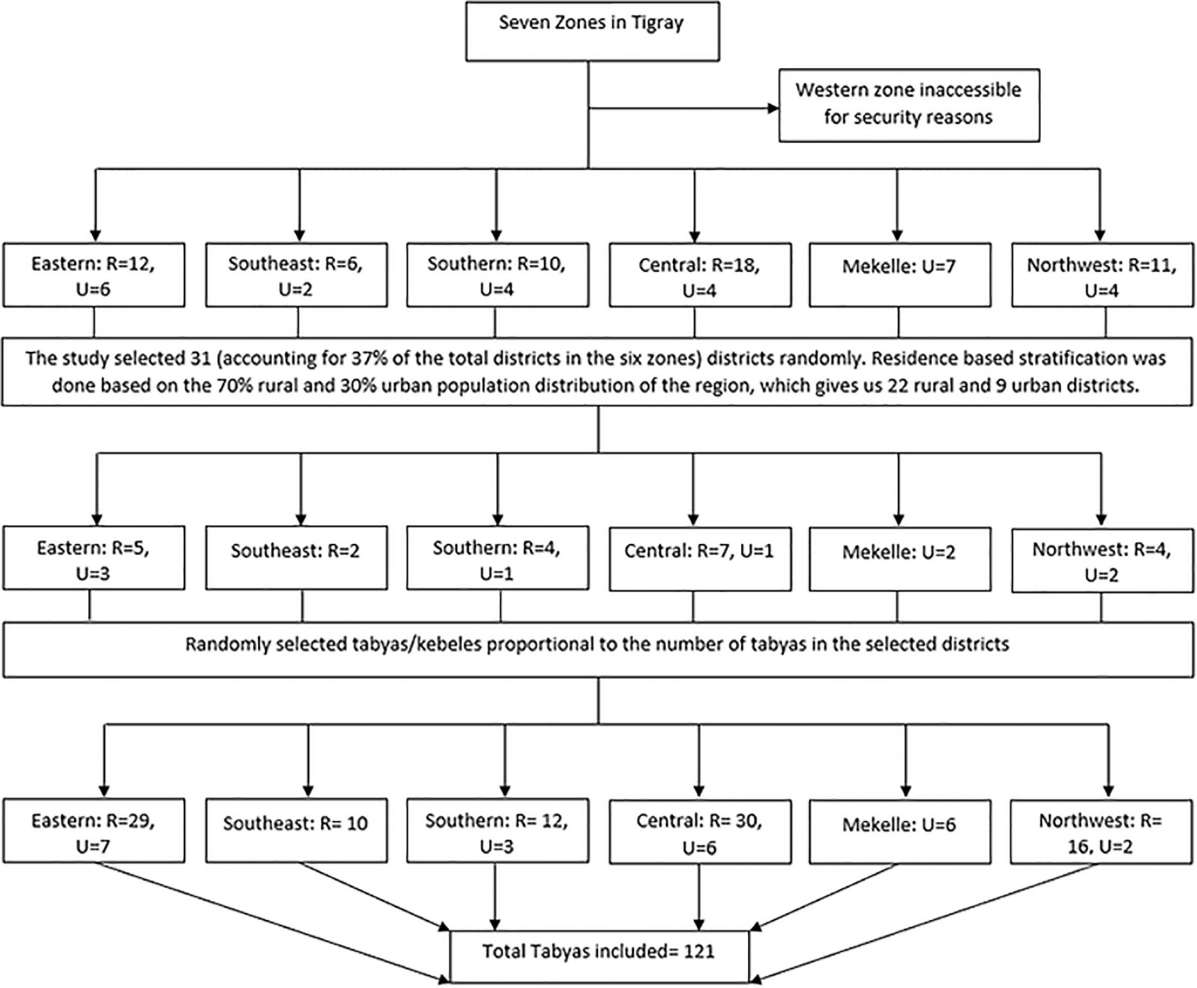
Sampling frame for the study.

In our effort to enhance the precision and accuracy of the maternal mortality ratio, we incorporated 37% of the districts into the survey. It’s important to note that this study constitutes a secondary analysis of our initial research. Therefore, for this analysis, we calculated power using Stata statistical software. We specified a fixed sample size of 832, an alpha level of 0.05, and a cluster number of 31. Additionally, the proportion of maternal deaths among women of reproductive age, taken as a key parameter for power calculation, was set at 0.30. Accordingly, the calculated power, indicating the study’s ability to detect true effects, was determined to be 0.92. We used a multistage stratified cluster sampling technique to select Tabiyas/Kebeles (the smallest administrative unit). First, the districts were stratified according to residence (urban versus rural) using the most recent proportion of urban residents in the region, which was found to be 30% [21]. Subsequently, 31 districts (22 rural and 9 urban) were randomly selected from the total 84 (57 in rural and 27 in urban settings) districts within the six zones to ensure representativeness. Within these 31 districts, 121 Tabiyas/Kebeles were selected based on the total number of Tabiyas in each district. The expected population size of each Tabiya is 5000–7000. The median number of Tabiyas among the selected districts was seven. Then random selection criteria were applied within each district as follows: three Tabiyas were selected for districts with fewer than seven Tabiyas, five for districts with seven to sixteen Tabiyas, and seven for districts with more than sixteen Tabiyas. All households in the 121 selected Tabiyas were evaluated using a screening tool, followed by VA for all deaths of women in the reproductive age group within the household (Fig 1).

### Data collection tools and procedures

The data collection tool used in this study was the standard 2022 WHO VA instrument, which incorporates the latest international classification of diseases, tenth revision (ICD-10). The tool was translated from English into the local language (Tigrigna) by bilingual health professionals to ensure accuracy and clarity. Then, it was translated back into English by independent bilingual health professionals. Following WHO VA recommendations, data were collected using an interviewer-administered questionnaire. The Open Data Kit (ODK) tool was used for data collection.

A total of 121 data collectors, 31 supervisors, and 6 data managers were recruited for this study. After a seven-day training period for data collectors and supervisors, a pretest was conducted on the final version of the questionnaire to ensure its validity. Data collection was conducted in two phases; first, all households were screened for any deaths of women in the reproductive age group. Subsequently, VA were conducted for all deceased women aged 12–49. Respondents to VA questions were carefully selected from family members or relatives, with priority given to the main caregiver who was with the deceased during the period leading up to death.

To maintain data quality, the supervisors reviewed the ODK and made daily copies of the backup data. Additionally, the supervisors controlled the distribution of households among the data collectors. The research team also conducted mid-data collection supervision to monitor progress.

### Data analysis

The data collected in this study was initially merged within the ODK platform before being exported to a comma-separated value (CSV) file using ODK Briefcase. Regional level preset HIV-Malaria mortality was considered “high” as per the 2022 WHO VA instrument recommendation for East African countries [22]. The dataset was then separated into two parts: screening data and VA data. The CSV file was subsequently imported into R software for analysis. Descriptive statistics were performed using frequency and percentage for categorical variables. As age was the sole continuous variable and exhibited a normal distribution, we utilized the mean with standard deviation (SD) as measures of central tendency and dispersion. VA data were processed using the InterVA–5.1 model, utilizing the OpenVA and CrossVA R software packages. The InterVA model uses probabilistic modelling to assign the underlying CoD [23]. These packages provided cause-specific mortality fractions (CSMF) for each case. The calculation of CSMF takes into consideration the likelihood probability associated with each cause of death. For instance, if we have a scenario with multiple deaths, the CSMF reflects the degree to which a particular cause contributed to these deaths. It is important to note that in certain instances, the software may produce error results or label cases as "undetermined" based on the reported symptoms and quality of data.

To determine the point estimates, confidence intervals, and standard errors for Global Burden of Disease (GBD) proportions, we used inverse probability weighting based on Tabiya selection. Districts were primary sampling units, with residence as the strata variable. The weight for Tabiyas was inversely calculated as the total number of Tabiyas in each district divided by the number of selected Tabiyas. This weighting accounted for varying Tabiya selection probabilities, ensuring accurate representation of GBD proportions. The linearized method estimated the variance-covariance matrix, providing robust population estimates considering the survey’s design complexities.

### Global burden of disease classification

To look at broad CoD categories, we aggregated the specific underlying CoD attributed to each death by GBD category. Unlike CSMF, which considers likelihood probabilities, the grouping in this context was determined based on the proportion of the primary CoD (cause1 in InterVA ascertainment).

- Group I. Infectious and parasitic diseases, and maternal causes, and malnutrition.
- Group II. Noncommunicable diseases and mental health conditions
- Group III. Injuries, including accidents, suicide, violence, and conflict

### Ethical approval

Ethical approval was obtained from the Ethics Review Committee of Mekelle University College of Health Sciences (IRB# 1963/2022). In addition, a support letter was acquired from the Tigray Regional Health Bureau. Following confirmation of the support letter by the regional health bureau, each district level health office provided additional approval for conducting the study. The objective of the survey was clarified to the person in charge of the household, and their verbal consent was obtained.

## Results

### Demographic characteristics

Out of the 189,087 households screened, 880 deaths of women of reproductive age were reported. However, only 832 were included in the study, as 48 (5.45%) of them were internally displaced women. The participants had a mean age of 37 years (SD = 9 years), with the majority (19.1%) falling within the age range 35–39 years. Among the participants, 650 (78.1%) resided in rural areas, and 563 (67.7%) were married. Regarding the level of education, 315 (38.1%) had not received any formal education, and nearly half (48.8%) were unemployed (Table 1).

**Table 1:** Characteristics of deceased women of reproductive age in Tigray, Northern Ethiopia, 2020–2022 (n = 832).

Characteristics	Frequency	Percent
Age in years [range 12–49]		
12–14	30	3.6
15–19	87	10.5
20–24	115	13.8
25–29	134	16.1
30–34	135	16.2
35–39	159	19.1
40–44	108	13.0
45–49	64	7.7
Residence, n (%)		
Urban	182	21.9
Rural	650	78.1
Marital status, n (%)		
Single/too young to be married	168	20.2
Married	563	67.7
Divorced	75	9.0
Life partner	12	1.4
Widowed	14	1.7
Educational status, n (%)		
No formal education	315	38.1
Primary school	265	32.0
Secondary school	148	17.9
Higher than secondary	99	12.0
Occupation, n (%)		
Homemaker	182	21.9
Employed	100	12.0
Unemployed	406	48.8
Student	68	8.2
Other	76	9.1

Note: The percentage in this table is not a survey-weighted percent.

### Broad causes of death

Among the three broad CoD based on the GBD classification, infectious, maternal, and nutritional disorders accounted for 354 (42.9%), followed by external CoD at 220 (26.4%) of primary CoD. Of the total 832 women of reproductive age deaths, 250 (30.0%) were maternal deaths. Two hundred (24.0%) deaths were attributed to communicable diseases. Cardiovascular and circulatory disorders top the group of noncommunicable disease (NCD) group, accounting for 84 (10.1%) deaths, and neoplasm-related deaths are the second leading subgroup of NCD at 67 (8.1%). Road traffic and other transport accidents caused 87 (10.5%) deaths (Table 2). As shown in S1 Fig, communicable and maternal disorders occurred predominantly in most districts according to the group-based classification of diseases.

**Table 2:** The three broad causes of death categories and subcategories among women of reproductive age death in Tigray, Northern Ethiopia, 2020–2022 (n = 832).

Causes of death	Frequency	Linearized Std. Error	Percent (95% CI)
**Communicable, maternal, and nutritional disorders**	**354**	**0.0187**	**42.9 (39.1, 46.7)**
Maternal deaths	250		30.0
Communicable diseases	200		24.0
**Noncommunicable diseases**	**192**	**0.0142**	**23.0 (20.2, 26.0)**
Cardiovascular and circulatory disorders	84		10.1
Neoplasms	67		8.1
Other NCDs	41		4.9
**External causes (Injuries)**	**220**	**0.0684**	**26.4 (23.1, 30.0)**
Road traffic and other transport accidents	87		10.5
Accidental drowning and poisoning	47		5.6
Other external causes	86		10.3

*Note: Some communicable diseases related CoD, which were also causes for maternal death are counted in both categories. Maternal death: Female deaths from any cause related to or aggravated by pregnancy or its management during pregnancy and childbirth or within 42 days after termination of pregnancy, regardless of the duration and site of pregnancy*. *CSMF: Cause Specific Mortality Fraction. Global Burden of Disease (GBD) classification was calculated by aggregating the specific underlying causes of death attributed to each death, grouping them into broad categories to examine the overall burden of disease. The figure for GBD is presented in proportion of deaths not CSMF. We computed confidence intervals (CI) and standard errors for the GBD primary CoD proportions using inverse probability weighting based on Tabiya selection*.

### Cause specific mortality fraction

One of the key findings of the current study was that HIV/AIDS-related deaths were the leading cause, accounting for 13.2% of all deaths. Obstetric hemorrhage (11.7%), other and unspecified cardiac diseases (6.6%), other transport accidents (6.5%), road traffic accidents (4.8%), tuberculosis (4.5%), and assault (3.9%) were the leading CoD following HIV/AIDS-related deaths. HIV/AIDS-related deaths comprised 29.9% of the communicable, maternal, and nutritional group, and surprisingly, 54.0% of all deaths related to communicable diseases were due to HIV/AIDS. Among this group, obstetric hemorrhage (27.7%) and pulmonary tuberculosis (10.2%) were the second and third leading CoD, respectively.

**Fig 2:**
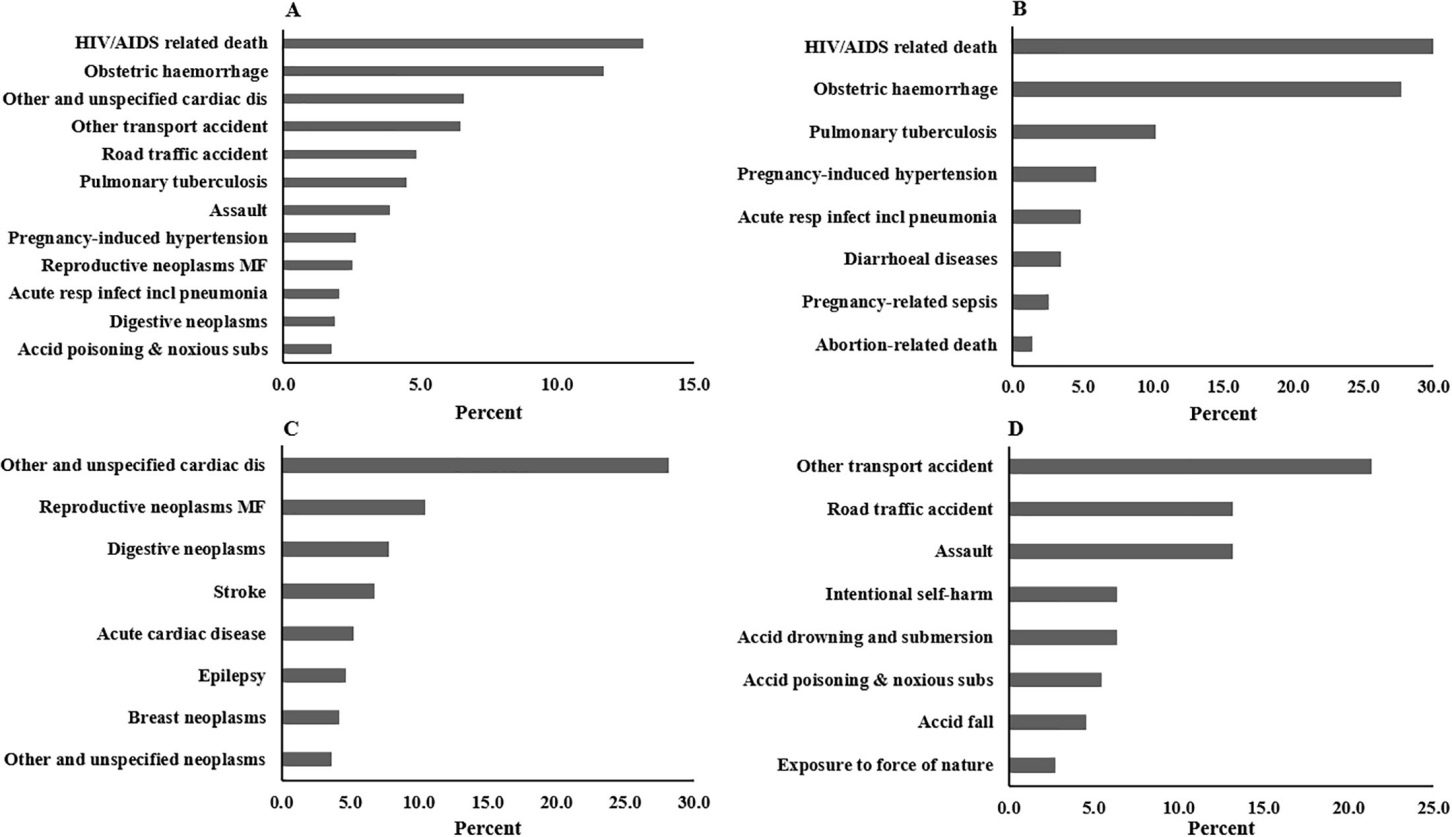
Cause-specific mortality fraction (CSMF) among women of reproductive age death in Tigray, Northern Ethiopia, 2020–2022 (n = 832). **A**) Overall CSMF, **B**) CSMF of communicable and maternal diseases group, **C)** CSMF of non-communicable diseases group, **D)** CSMF of external causes or injuries. Note that the survey weighting we used may not directly impact the assignment of causes to individual deaths. This applies specifically when using the verbal autopsy tool.

In the group of noncommunicable diseases, other and unspecified cardiac diseases (28.1%) were the major CoD. Reproductive neoplasms MF (10.4%), digestive neoplasms (7.8%), stroke (6.8%), and acute cardiac disease (5.2%) ranked as the second to fifth major causes, respectively. Among the categories of external causes, other transport accidents (21.4%), road traffic accidents (13.2%), assault (13.2%), intentional self-harm (6.4%), and accidental drowning and submersion (6.4%) were the five leading CoD (Fig 2). According to the CSMF, 15.9% CoD were classified as “undetermined”. A list of the CSMFs of all underlying causes is presented in S1 Table.

### Association between causes of death and age

**Fig 3:**
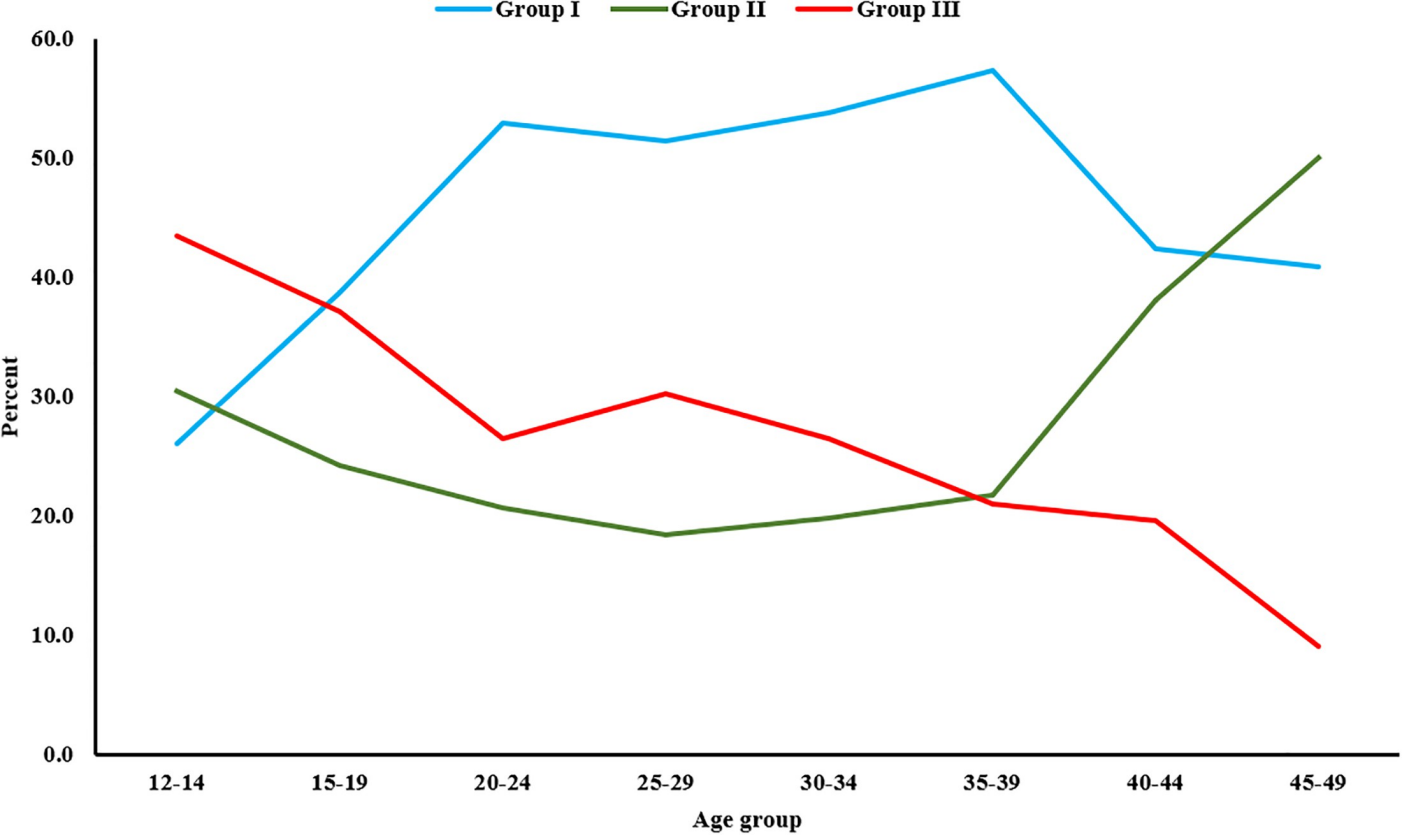
Group-based Cause-Specific Mortality Fraction (CSMF) versus age group among women of reproductive age death in Tigray, Northern Ethiopia, 2020–2022 (n = 832). Group I: Communicable, maternal, and nutritional disorders, Group II: Non-communicable diseases, and Group III: External causes (Injuries). Note that the survey weighting we used may not directly impact the assignment of causes to individual deaths. This applies specifically when using the verbal autopsy tool.

The group-based classification of causes versus the distribution of age categories revealed a slight increasing trend for infectious and maternal causes in the first six data points, followed by a decrease in the middle and a drop at the end of the two observations. In group two (communicable group), the graph initially showed a slight decrement in the proportion of communicable diseases as the age increased (from 12 to 29 years), but then exhibited a spike in the next four age groups. In the external causes grouping, there was a clear decreasing trend in external causes as age increased (Fig 3).

## Discussion

This study examined CoD among women of reproductive age during the armed conflict in Tigray, Ethiopia. The classification of diseases according to groups revealed that infectious diseases and maternal causes were dominant, accounting for 42.9% of all deaths. External causes exceeded infectious disease causes. Thirty percent of women died of pregnancy and childbirth. HIV / AIDS has emerged as the leading CoD, accounting for 13.2% of all deaths and 54.0% of communicable diseases. Among external causes, assault, which directly resulted from the war, accounted for 13.2%. Other and unspecified cardiac diseases were the third leading CoD among the specific CoD.

We found that the leading CoD among women of reproductive age during the armed conflict in Tigray, Ethiopia, were infectious diseases and maternal factors, according to the GBD classification. This is consistent with previous studies conducted in peaceful settings, which have consistently found that infectious diseases and pregnancy-related causes are the main killers among women in developing countries [24–27]. In some studies, even infectious disease related deaths alone outnumber NCDs and external causes combined [25,26]. Although infectious diseases were dominant even in non-conflict settings, research shows that the impact of infectious diseases usually increases after armed conflicts, mostly due to health system disruption [28,29]. In addition, due to the impact of the war on Tigray’s health system, patients with chronic diseases have discontinued their regular check-ups and lost access to necessary medications [11,30]. This situation has potentially led to an increase in deaths related to infectious diseases.

Our findings showed that external causes claimed more lives than infectious diseases and NCDs with assault accounting for 13.2% of them. In developing countries such as Ethiopia the usual ranking of GBD classification flows as infectious diseases, NCDs and then external causes. In a Health and Demographic Surveillance System (HDSS) study conducted in non-conflict setting in Tigray, for example, external cause were the third largest cause after infectious diseases and NCDs, and assault was not on the list of CoD at all [26]. This shows that the war in Tigray has shifted the ranking of the main CoD, with assault being directly influenced by the armed conflict. In addition, other external causes such as inflicted self-harm and accidental falls may be exacerbated by the war.

In this study, maternal deaths constituted 30.0% of deaths among women of reproductive age. This figure was quite higher than the reports from Ethiopia, Nigeria, and Iran which were conducted in conflict-free periods [26,27,31,32]. The reason for the higher proportion of maternal deaths during wartime could be because pregnant women are more vulnerable during conflicts, which is linked to significant and long-lasting increases in child-bearing age and maternal mortality [9,33]. During the war in Tigray, there was an extreme increase in home delivery [14], which is likely one of the reasons for the increase in pregnancy related deaths. Details of causes of maternal death from this study are reported and discussed elsewhere [20].

Our research revealed that the primary CoD among women of reproductive age during the armed conflict in Tigray, Ethiopia, was HIV/AIDS. Although their data were based on a HDSS, three studies conducted in a peaceful setting in Ethiopia showed a similar finding to ours that HIV/AIDS was the primary CoD [25,26,32]. HIV/AIDS has been the number one killer among women of reproductive age for a long time now [2,3,34]. The fact that HIV/AIDS is the main CoD is not unexpected, as a recent reports examining HIV care in Tigray revealed a significant increase in the number of patients lost to follow-up and dropped out after the conflict, resulting in a reduction of up to 76.4% in antiretroviral therapy (ART) enrolment and 80.0% in the overall follow-up rate [30,35]. The fact that over three-quarters of HIV/AIDS patients from rural and semiurban areas of Tigray did not receive their treatments [30] clearly demonstrates that these patients were more vulnerable to developing opportunistic infections and experiencing fatalities. Furthermore, even about five months after the peace agreement between the warring parties, in May 2023, the Tigray health bureau reported that the status of at least 13,000 known HIV/AIDS patients, who were enrolled in a care, was unknown [36].

While 10.2% of the women died of cardiovascular and circulatory disorders, other and unspecified cardiac diseases were the third leading CoD, comprising more than half of the cardiovascular and circulatory disorders. Although cardiac diseases in general are prevalent and among the leading top CoD in Ethiopia [37,38], report show that other and unspecified cardiac diseases may have low positive predictive value against medical review [39].

While the fact that the study used globally accepted validated WHO VA tool to ascertain CoD in a community where reliable vital statistics registration is weak is its strength, the study has several limitations. First, because the study was a post hoc analysis of maternal death study, data on the total number of individuals in the reproductive age group within each household was not available, which made it impossible to calculate the death rate among women of reproductive age. Second, the data was collected using the 2022 WHO VA instrument. However, as InterVA software for this questionnaire has not yet been developed, we used the InterVA-5.1 model that was designed for 2016 WHO VA tool. Third, the persisting conflict and humanitarian blockade in the region have caused the displacement of households, making them potentially inaccessible for surveys. This, in turn, might have resulted in a reduction in the number of reported deaths. Fourth, excluding the western zone of Tigray from our study, due to security reasons, is another important limitation. There have been reports of severe atrocities occurring in the Tigray region, with even more incidents in the western zone [40,41]. Consequently, it is likely that the causes and the number of deaths in this zone differ from those in the areas we have included in our study.

## Conclusions

In the current study infectious disease related deaths dominated the group based GBD classification with 42.6% of total deaths. Pregnancy and childbirth-related causes comprised 30.0% of the overall deaths. HIV/AIDS was the number one killer. Assault was the second leading CoD among external CoD. The findings of the study indicate that the occurrence of deaths due to infectious CoD, such as HIV/AIDS, and uncommon CoD such as assault, as well as a significant number of maternal deaths in Tigray, Ethiopia, are highly likely increased as a consequence of the war in the region. This emphasizes the immediate need for focused interventions and allocation of resources to tackle these problems and give priority to maternal health in Tigray.

## Supporting information

S1 Fig Group-based category for each district, among women of reproductive age death in Tigray, Northern Ethiopia, 2020–2022 (n = 832). Group I: Communicable, maternal, and nutritional disorders, Group II: Non-communicable diseases, and Group III: External causes (Injuries). This classification is presented based on the proportion of primary CoD not CSMF. The percentage in this figure is not a survey-weighted percent.
(DOCX)

S1 Table List of all causes of death among women of reproductive age death in Tigray, Northern Ethiopia, 2020–2022 (n = 832). (DOCX)

S1 Dataset (DTA)
