# Supplementary material for: Causes of death among women of reproductive age during the war in Tigray, Ethiopia
Source: PLoS One. 2024 Mar 13;19(3):e0299650. doi: 10.1371/journal.pone.0299650 (PMC10936770; doi:10.1371/journal.pone.0299650)
Supplement: S1 Fig — Group I: Communicable, maternal, and nutritional disorders, Group II: Non-communicable diseases, and Group III: External causes (Injuries). This classification is presented based on the proportion of primary CoD not CSMF. The percentage in this figure is not a survey-weighted percent. (DOCX) [file pone.0299650.s001.docx]

**

*S1 Fig. Group-based category for each district, among women of reproductive age death in Tigray, Northern Ethiopia, 2020-2022 (n=832). Group I: Communicable, maternal, and nutritional disorders, Group II:* *Non-communicable diseases, and Group III:* *External causes (Injuries). This classification is presented based on the proportion of primary CoD not CSMF. The percentage in this figure is not a survey-weighted percent.*
