# Supplementary material for: Causes of death among women of reproductive age during the war in Tigray, Ethiopia
Source: PLoS One. 2024 Mar 13;19(3):e0299650. doi: 10.1371/journal.pone.0299650 (PMC10936770; doi:10.1371/journal.pone.0299650)
Supplement: S1 Table — (DOCX) [file pone.0299650.s002.docx]

*S1 Table: List of all causes of death among women of reproductive age death in Tigray, Northern Ethiopia, 2020-2022 (n=832)*

| Cause of Death | CSMF (%) |
| --- | --- |
| HIV/AIDS related death | 13.16 |
| Obstetric haemorrhage | 11.72 |
| Other and unspecified cardiac disease | 6.59 |
| Other transport accident | 6.45 |
| Road traffic accident | 4.84 |
| Pulmonary tuberculosis | 4.49 |
| Assault | 3.91 |
| Pregnancy-induced hypertension | 2.63 |
| Reproductive neoplasms MF | 2.54 |
| Acute resp infect including pneumonia | 2.04 |
| Digestive neoplasms | 1.89 |
| Accidental poisoning & noxious subs | 1.77 |
| Intentional self-harm | 1.77 |
| Accidental drowning and submersion | 1.65 |
| Stroke | 1.57 |
| Diarrhoeal diseases | 1.49 |
| Pregnancy-related sepsis | 1.34 |
| Acute cardiac disease | 1.23 |
| Accidental fall | 1.17 |
| Epilepsy | 1.14 |
| Breast neoplasms | 1.09 |
| Other and unspecified neoplasms | 0.94 |
| Liver cirrhosis | 0.77 |
| Exposure to force of nature | 0.70 |
| Respiratory neoplasms | 0.69 |
| Abortion-related death | 0.68 |
| Renal failure | 0.65 |
| Acute abdomen | 0.56 |
| Contact with venomous plant/animal | 0.53 |
| Other and unspecified infect disease | 0.51 |
| Sickle cell with crisis | 0.50 |
| Other and unspecified NCD | 0.48 |
| Malaria | 0.47 |
| Accidental expos to smoke fire & flame | 0.38 |
| Other and unspecified maternal CoD | 0.37 |
| Diabetes mellitus | 0.34 |
| Meningitis and encephalitis | 0.24 |
| Obstructed labour | 0.20 |
| Anaemia of pregnancy | 0.16 |
| Sepsis (non-obstetric) | 0.12 |
| Ectopic pregnancy | 0.11 |
| Haemorrhagic fever (non-dengue) | 0.08 |
| Oral neoplasms | 0.07 |
| Ruptured uterus | 0.05 |
| Severe malnutrition | 0.05 |
| Undetermined | 15.9 |

*CSMF: Cause Specific Mortality Fraction. Note that the survey weighting we used may not directly impact the assignment of causes to individual deaths. This applies specifically when using the verbal autopsy tool.*
